# Supplementary material for: Effects of dietary polyphenol curcumin supplementation on metabolic, inflammatory, and oxidative stress indices in patients with metabolic syndrome: a systematic review and meta-analysis of randomized controlled trials
Source: Front Endocrinol (Lausanne). 2023 Jul 14;14:1216708. doi: 10.3389/fendo.2023.1216708 (PMC10376715; doi:10.3389/fendo.2023.1216708)
Supplement: Supplementary file 1 [file DataSheet_1.docx]

**Pubmed:**

("metabolic syndrome"[MeSH Terms] OR ("metabolic syndrome"[Title/Abstract] OR "Metabolic Syndromes"[Title/Abstract] OR "syndrome metabolic"[Title/Abstract] OR "syndromes metabolic"[Title/Abstract] OR "Metabolic Syndrome X"[Title/Abstract] OR "Insulin Resistance Syndrome X"[Title/Abstract] OR "syndrome x metabolic"[Title/Abstract] OR "syndrome x insulin resistance"[Title/Abstract] OR "Metabolic X Syndrome"[Title/Abstract] OR "syndrome metabolic x"[Title/Abstract] OR "x syndrome metabolic"[Title/Abstract] OR "Dysmetabolic Syndrome X"[Title/Abstract] OR "Reaven Syndrome X"[Title/Abstract] OR "syndrome x reaven"[Title/Abstract] OR "Metabolic Cardiovascular Syndrome"[Title/Abstract] OR "cardiovascular syndrome metabolic"[Title/Abstract] OR "syndrome metabolic cardiovascular"[Title/Abstract] OR "Cardiometabolic Syndrome"[Title/Abstract] OR "Cardiometabolic Syndromes"[Title/Abstract] OR "syndrome cardiometabolic"[Title/Abstract] OR "Insulin Resistance Syndrome X"[Title/Abstract] OR "insulin resistance"[Title/Abstract] OR "national cholesterol education program adult treatment panel iii"[Title/Abstract] OR "nutritional and metabolic diseases"[Title/Abstract] OR "metabolic diseases"[Title/Abstract] OR "plurimetabolic syndrome"[Title/Abstract] OR "atherothrombogenic syndrome"[Title/Abstract] OR "syndrome x plus"[Title/Abstract] OR "deadly quartet"[Title/Abstract] OR "metsyn"[Title/Abstract] OR "obesity syndrome"[Title/Abstract] OR "hypertriglyceridemic waist"[Title/Abstract])) AND ("Curcumin"[MeSH Terms] OR ("Curcumin"[Title/Abstract] OR "Turmeric Yellow"[Title/Abstract] OR "yellow turmeric"[Title/Abstract] OR "Curcumin Phytosome"[Title/Abstract] OR "Diferuloylmethane"[Title/Abstract] OR "curcuminoid"[Title/Abstract] OR "Curcuma"[Title/Abstract] OR "curcuminoid supplement"[Title/Abstract] OR "curcumin extract"[Title/Abstract]) OR ("Curcuma"[MeSH Terms] OR ("Curcuma"[Title/Abstract] OR "Curcumas"[Title/Abstract] OR "Curcuma zedoaria"[Title/Abstract] OR "zedoaria zedoary"[Title/Abstract] OR "Curcuma longa"[Title/Abstract] OR "longa curcuma"[Title/Abstract] OR "Tumeric"[Title/Abstract] OR "Turmeric"[Title/Abstract] OR "Turmerics"[Title/Abstract]))) AND ("randomized controlled trial"[Publication Type] OR "randomized"[Title/Abstract] OR "placebo"[Title/Abstract] OR "RCTs"[Title/Abstract])

**Web of Science:**

4

#3 AND #2 AND #1

151

添加到检索式



3

TS=("randomized controlled trial” OR "randomized" OR "placebo" OR "RCTs" )

1040568

添加到检索式



2

TS=("curcumin” OR "Turmeric Yellow" OR "Yellow, Turmeric" OR "Curcumin Phytosome" OR "Diferuloylmethane" OR "curcuminoid" OR "curcuma" OR "curcuminoid supplement" OR "curcumin extract" OR "Curcuma" OR "Curcumas" OR "Curcuma zedoaria" OR "zedoaria, Zedoary" OR "Curcuma longa" OR "longa, Curcuma" OR "Tumeric" OR "Turmeric" OR "Turmerics")

34927

添加到检索式



1

TS=("Metabolic syndrome” OR "Metabolic Syndromes" OR "Syndrome, Metabolic" OR "Syndromes, Metabolic" OR "Metabolic Syndrome X" OR "Insulin Resistance Syndrome X" OR "Syndrome X, Metabolic" OR "Syndrome X, Insulin Resistance" OR "Metabolic X Syndrome" OR "Syndrome, Metabolic X" OR "X Syndrome, Metabolic" OR "Dysmetabolic Syndrome X" OR "Reaven Syndrome X" OR "Syndrome X, Reaven" OR "Metabolic Cardiovascular Syndrome" OR "Cardiovascular Syndrome, Metabolic" OR "Syndrome, Metabolic Cardiovascular" OR "Cardiometabolic Syndrome" OR "Cardiometabolic Syndromes" OR "Syndrome, Cardiometabolic" OR "insulin resistance syndrome x" OR "insulin resistance" OR "national cholesterol education program adult treatment panel iii" OR "nutritional and metabolic diseases" OR "metabolic diseases" OR "plurimetabolic syndrome" OR "atherothrombogenic syndrome" OR "syndrome x plus" OR "deadly quartet" OR "metsyn" OR "dysmetabolic syndrome" OR "obesity syndrome" OR "hypertriglyceridemic waist")

257728

**Cochrane Library:**


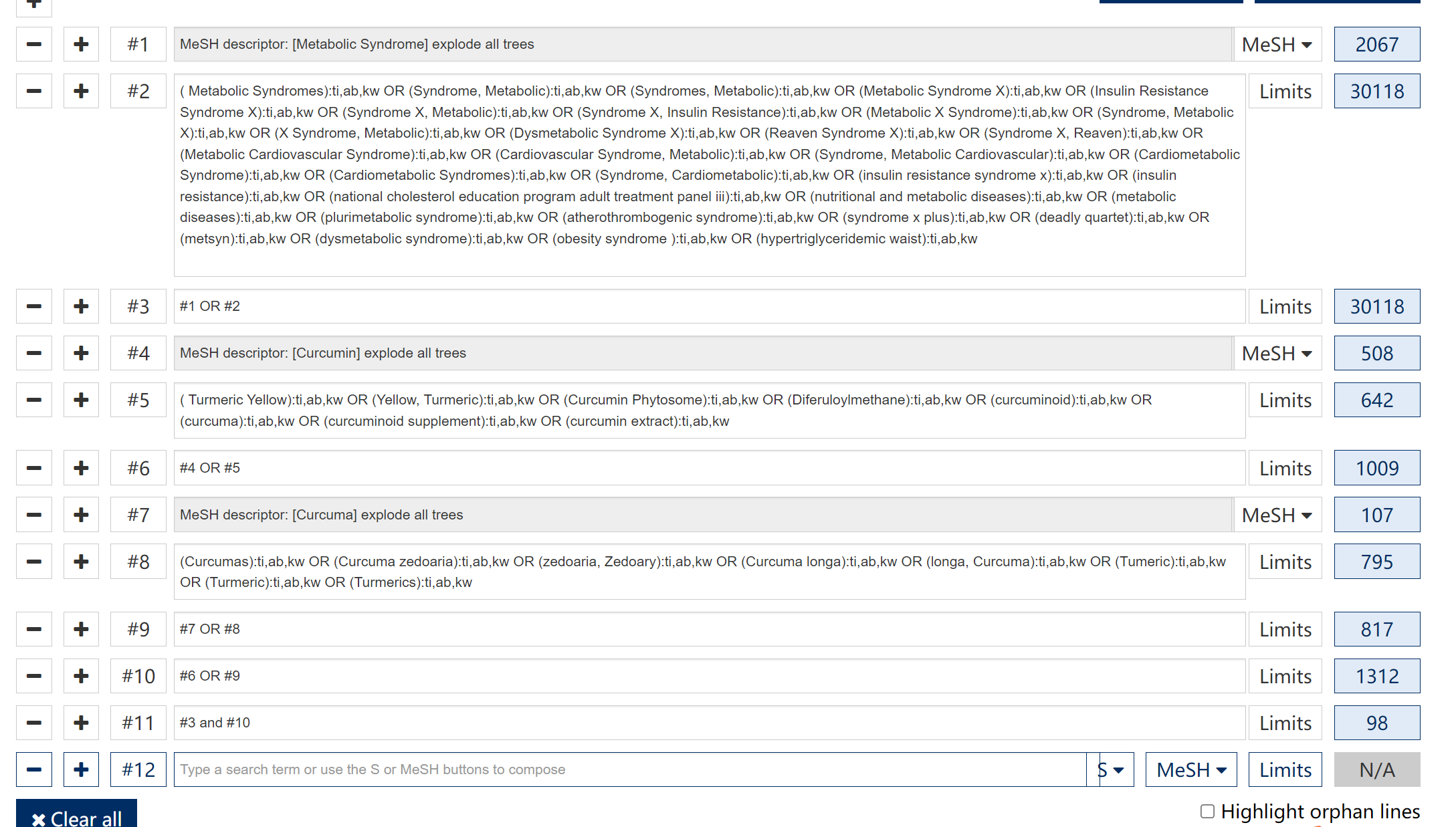


**CBM:**

**
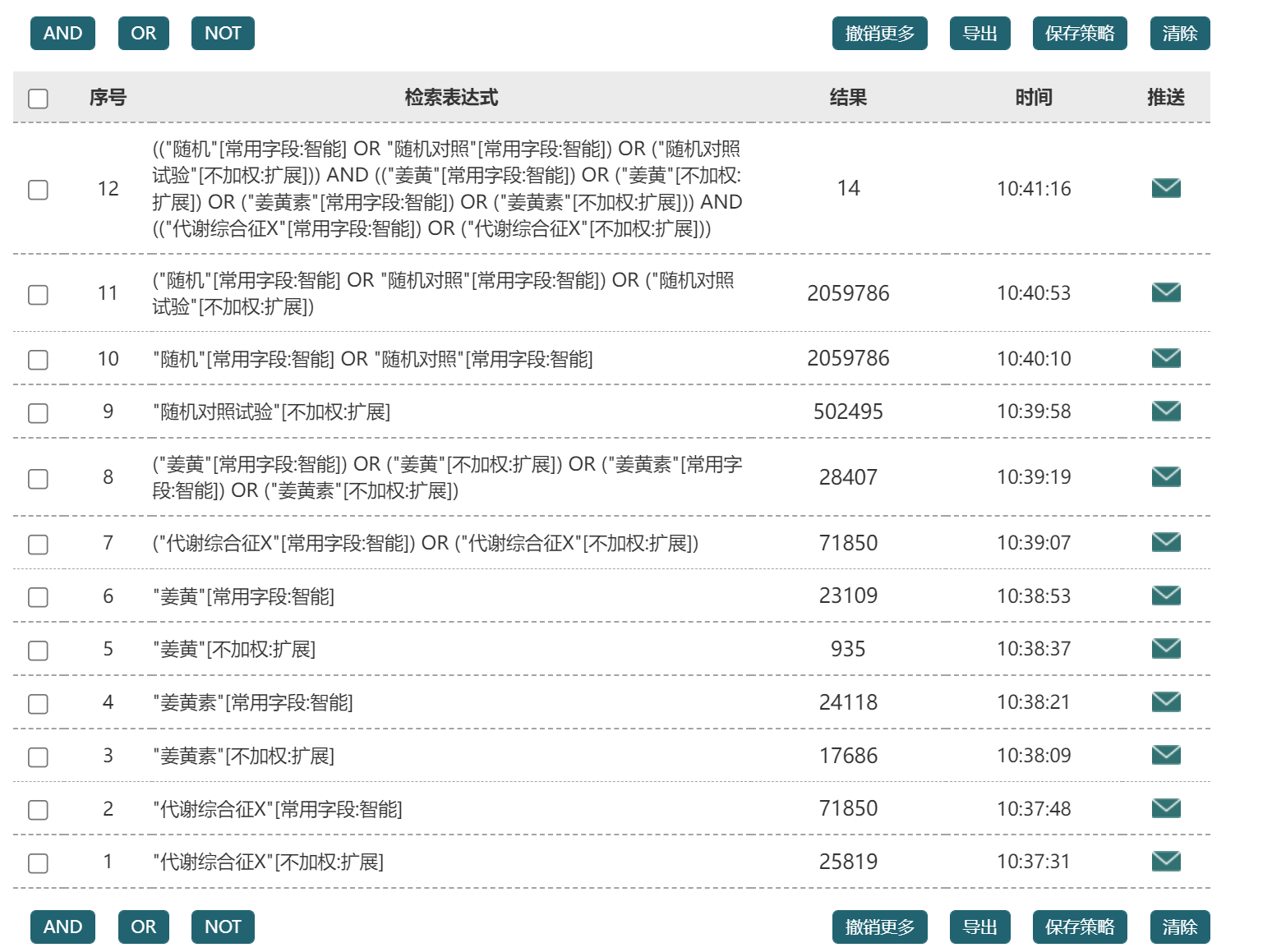
**

**EMBASE:**

**SCOPUS:**

TITLE-ABS-KEY ( "metabolic syndrome" OR "metabolic syndromes" OR "syndrome, metabolic" OR "syndromes, metabolic" OR "metabolic syndrome x" OR "insulin resistance syndrome x" OR "syndrome x, metabolic" OR "syndrome x, insulin resistance" OR "metabolic x syndrome" OR "syndrome, metabolic x" OR "x syndrome, metabolic" OR "dysmetabolic syndrome x" OR "reaven syndrome x" OR "syndrome x, reaven" OR "metabolic cardiovascular syndrome" OR "cardiovascular syndrome, metabolic" OR "syndrome, metabolic cardiovascular" OR "cardiometabolic syndrome" OR "cardiometabolic syndromes" OR "syndrome, cardiometabolic" OR "insulin resistance syndrome x" OR "insulin resistance" OR "national cholesterol education program adult treatment panel iii" OR "nutritional and metabolic diseases" OR "metabolic diseases" OR "plurimetabolic syndrome" OR "atherothrombogenic syndrome" OR "syndrome x plus" OR "deadly quartet" OR "metsyn" OR "dysmetabolic syndrome" OR "obesity syndrome" OR "hypertriglyceridemic waist" ) AND TITLE-ABS-KEY ( "curcumin" OR "turmeric yellow" OR "yellow, turmeric" OR "curcumin phytosome" OR "diferuloylmethane" OR "curcuminoid" OR "curcuma" OR "curcuminoid supplement" OR "curcumin extract" OR "curcuma" OR "curcumas" OR "curcuma zedoaria" OR "zedoaria, zedoary" OR "curcuma longa" OR "longa, curcuma" OR "tumeric" OR "turmeric" OR "turmerics" ) AND TITLE-ABS-KEY ("randomized controlled trial" OR "randomized" OR "placebo" OR "rcts" )
