# Supplementary figures and images for: Effects of dietary polyphenol curcumin supplementation on metabolic, inflammatory, and oxidative stress indices in patients with metabolic syndrome: a systematic review and meta-analysis of randomized controlled trials
Source: Front Endocrinol (Lausanne). 2023 Jul 14;14:1216708. doi: 10.3389/fendo.2023.1216708 (PMC10376715; doi:10.3389/fendo.2023.1216708)

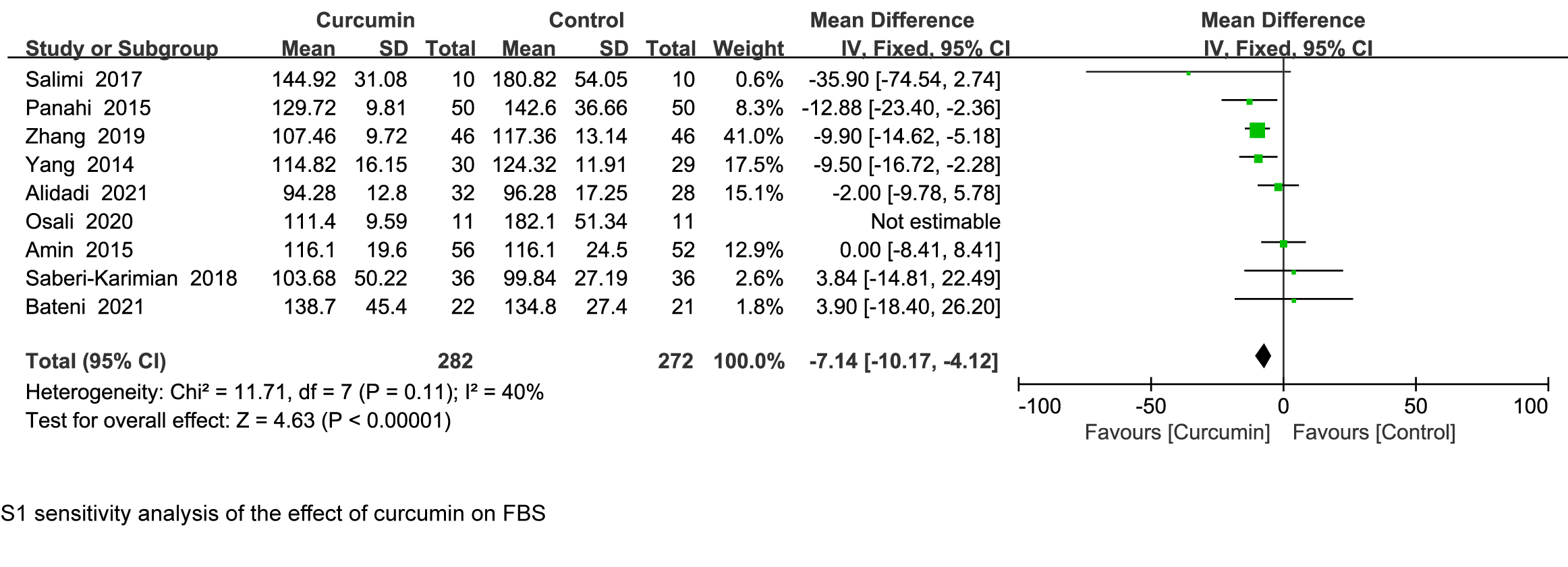

Supplement: Supplementary file 2 [file Image_1.tif]

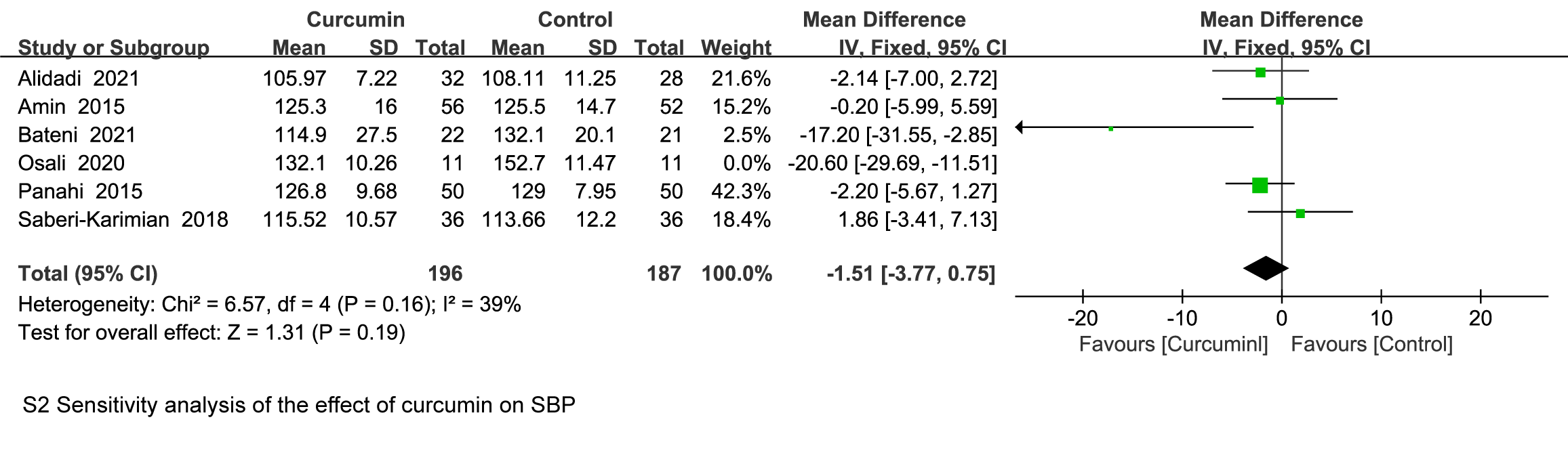

Supplement: Supplementary file 3 [file Image_2.tif]

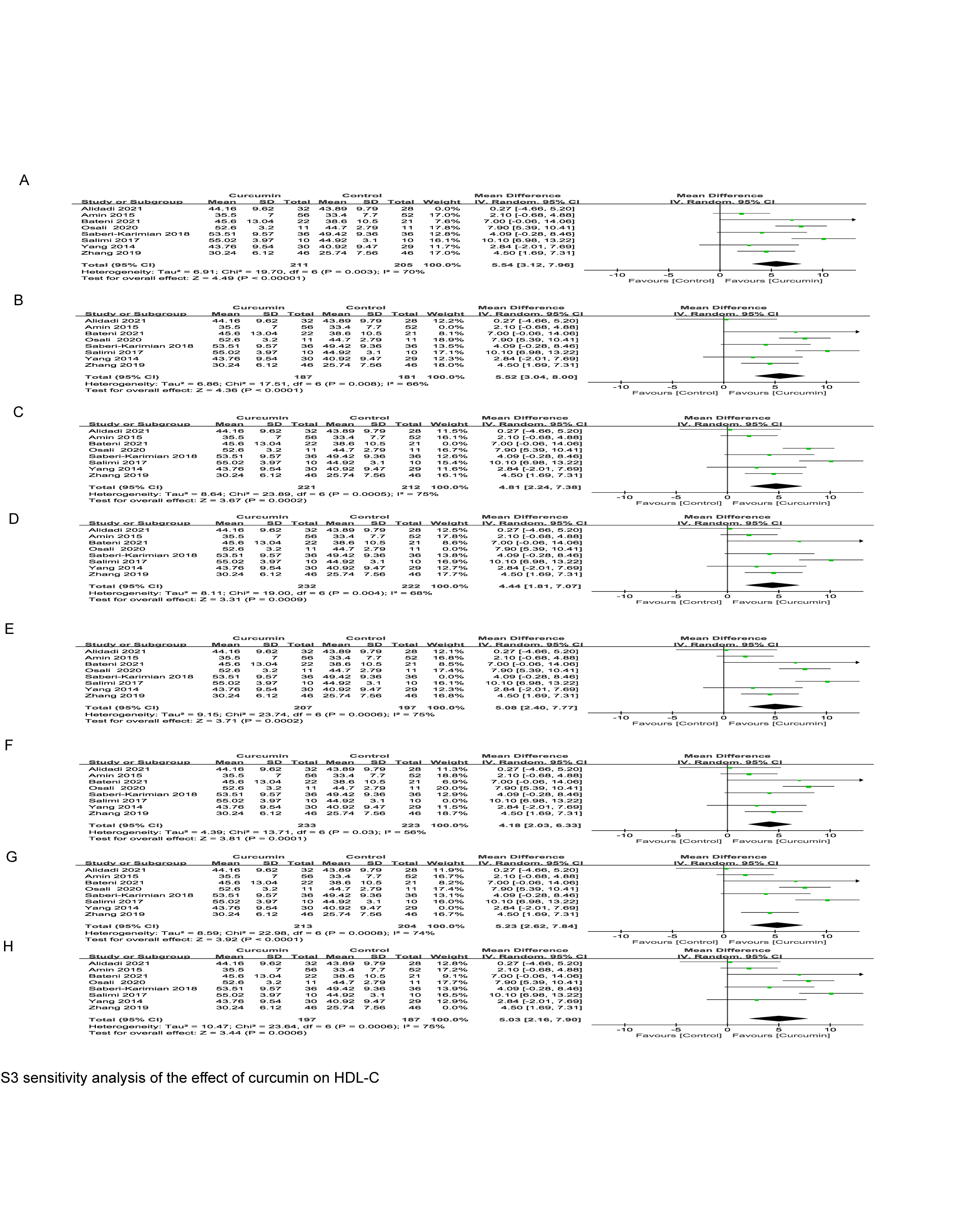

Supplement: Supplementary file 4 [file Image_3.tif]

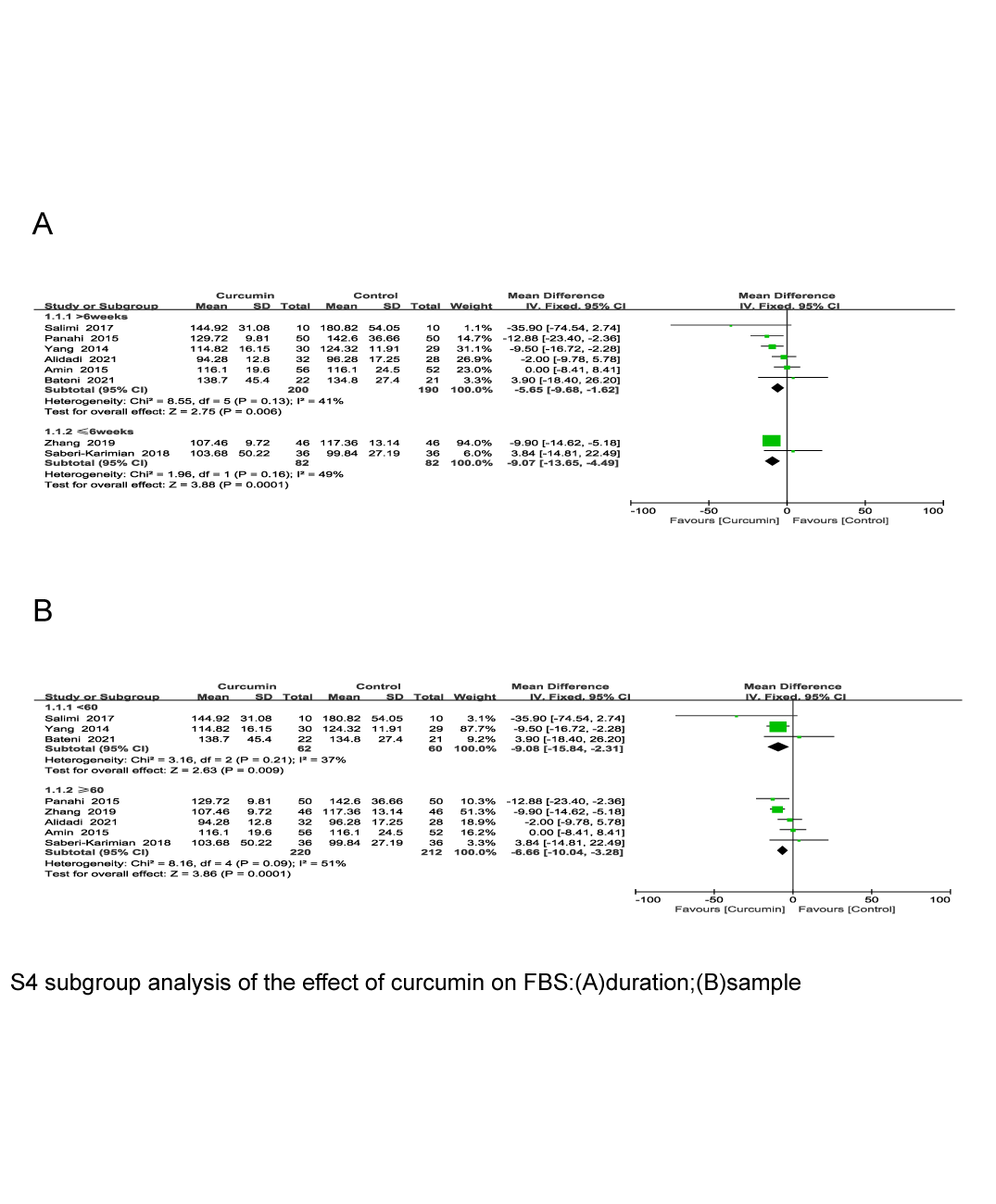

Supplement: Supplementary file 5 [file Image_4.tif]

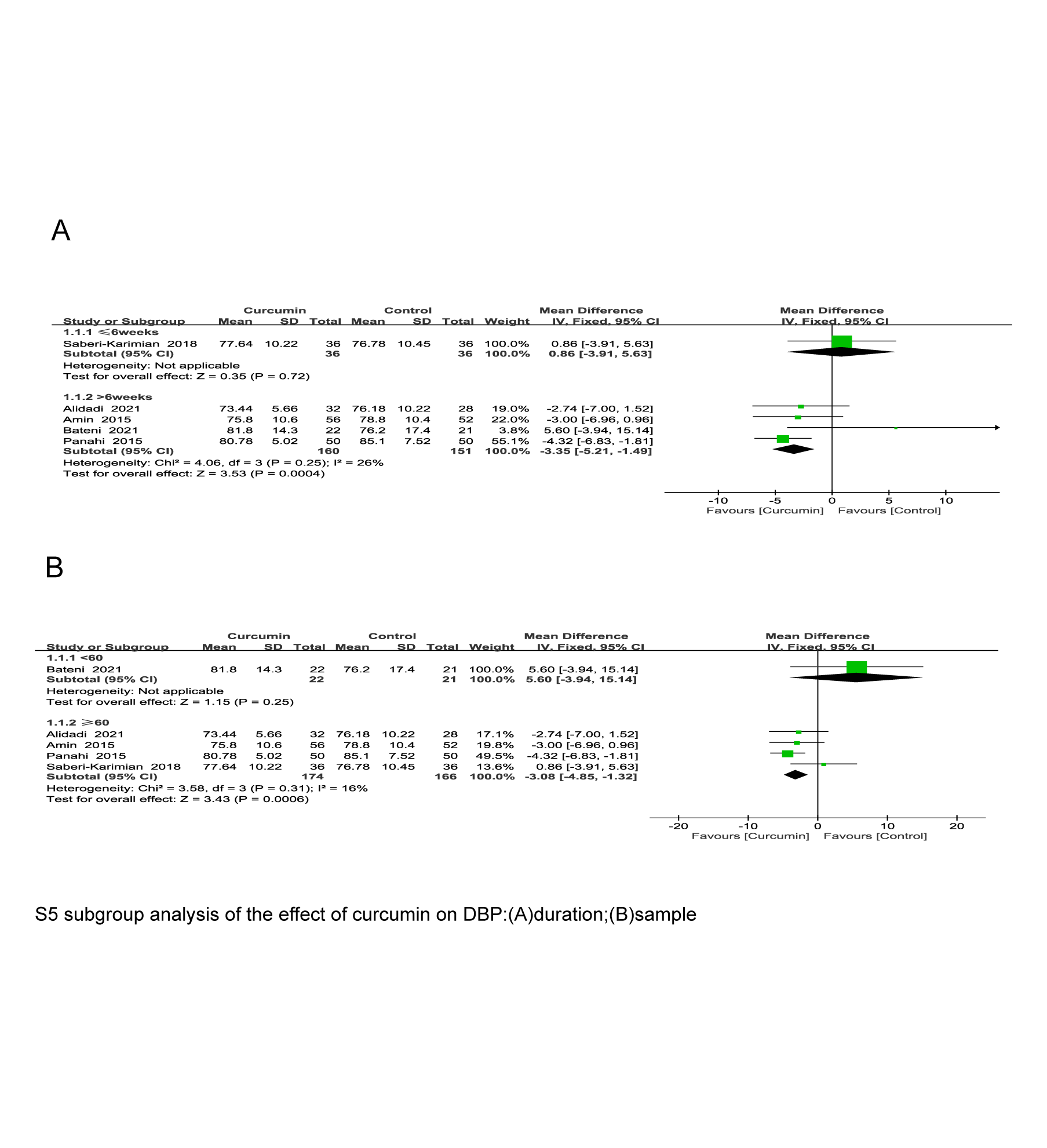

Supplement: Supplementary file 6 [file Image_5.tif]

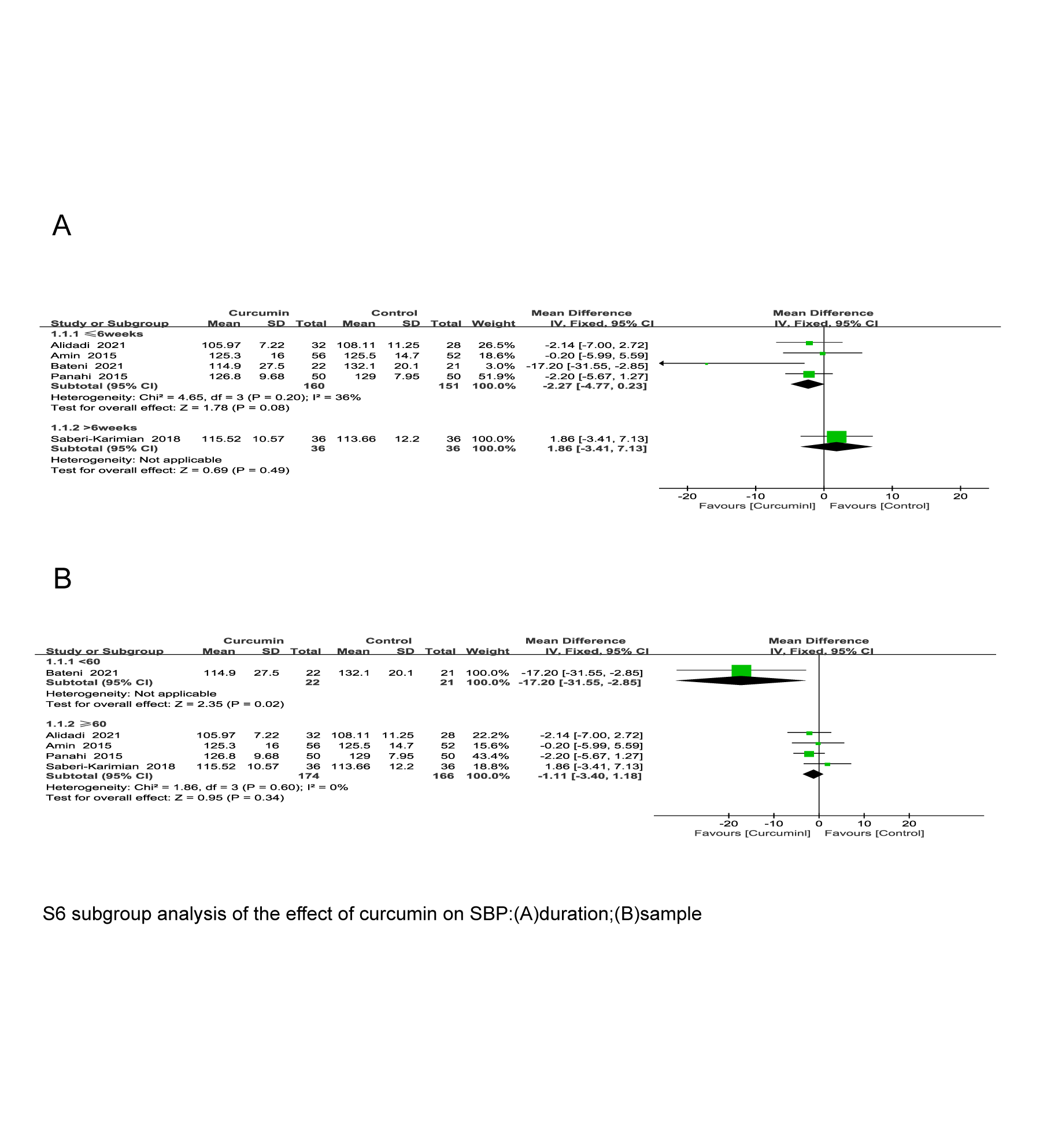

Supplement: Supplementary file 7 [file Image_6.tif]

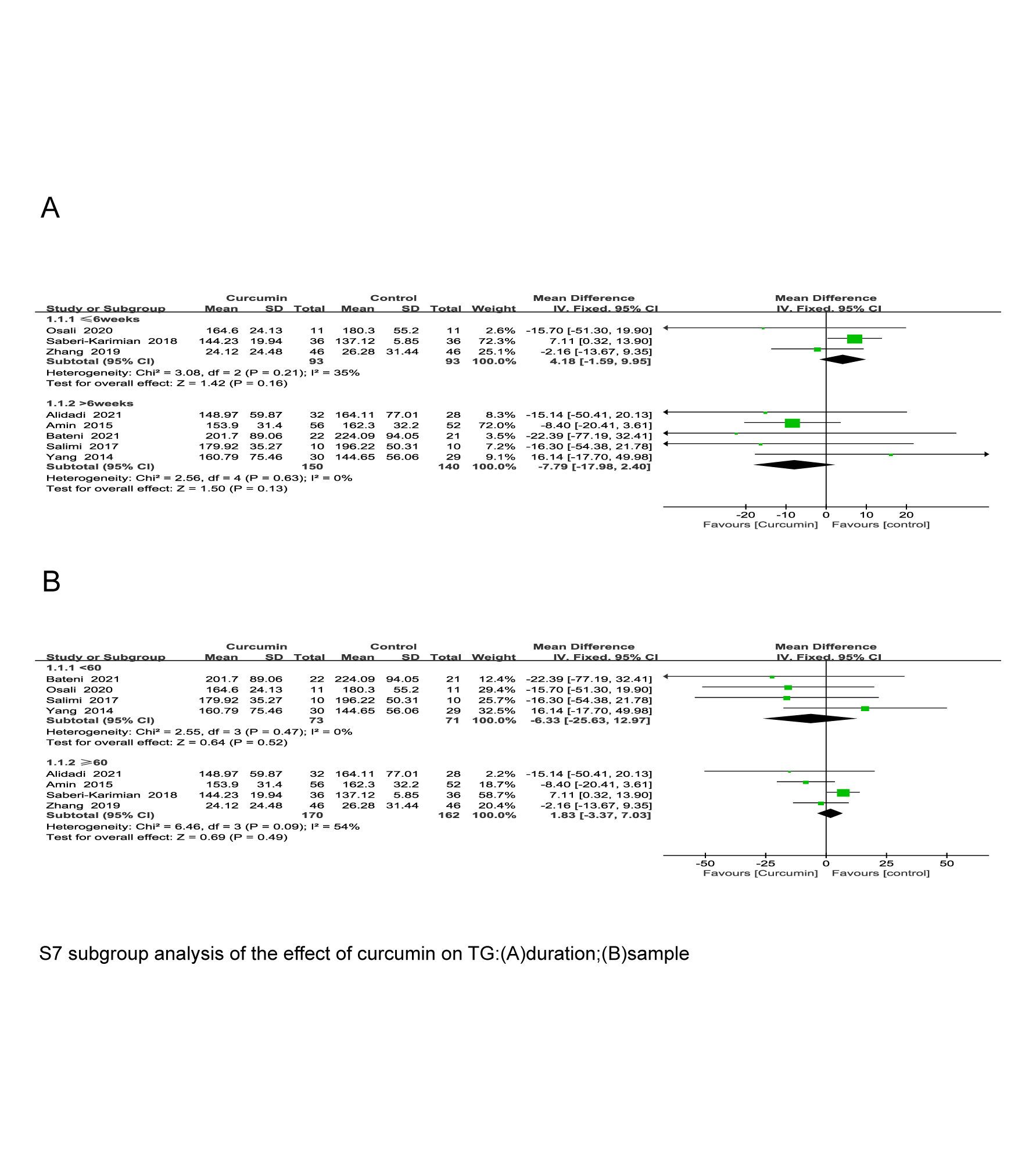

Supplement: Supplementary file 8 [file Image_7.tif]

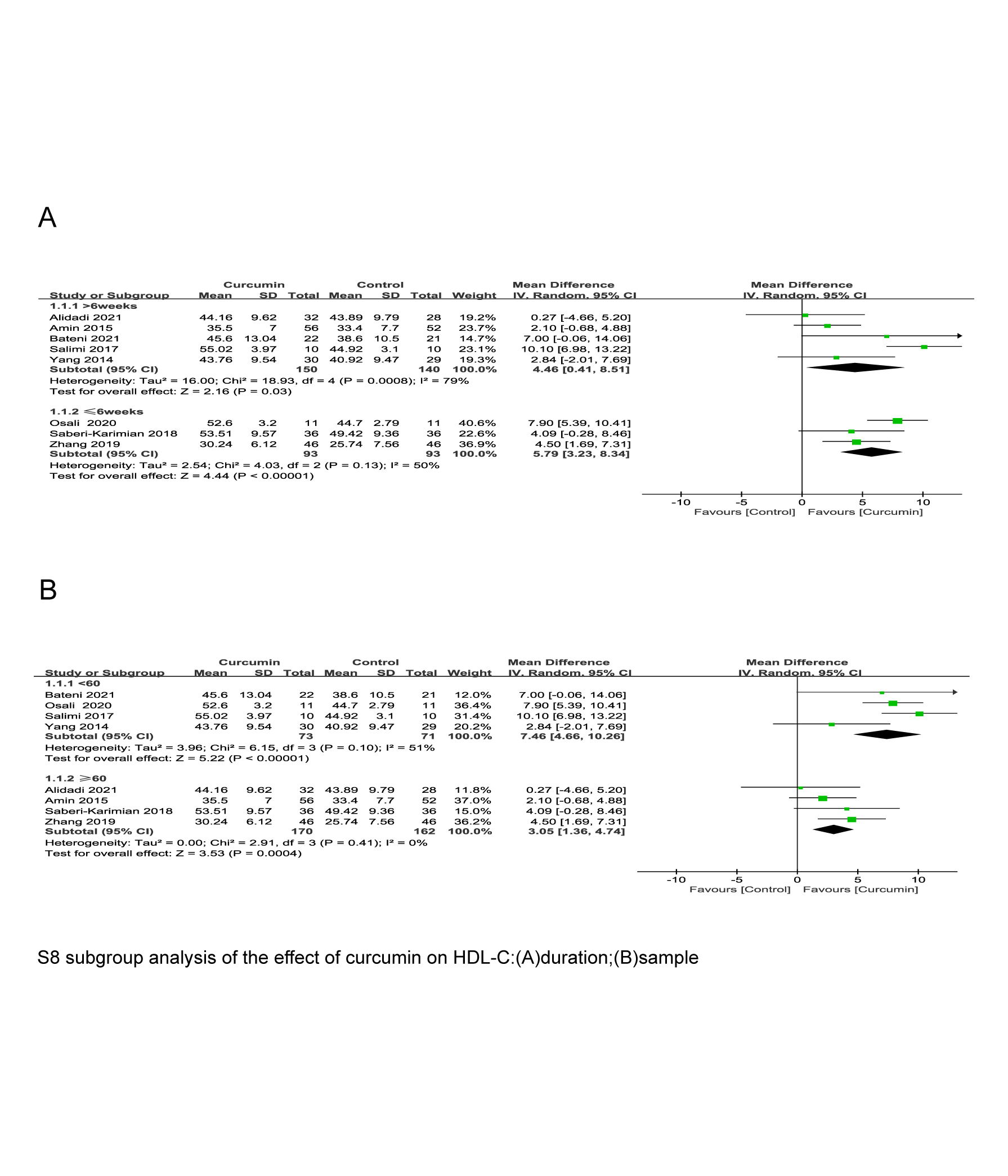

Supplement: Supplementary file 9 [file Image_8.tif]

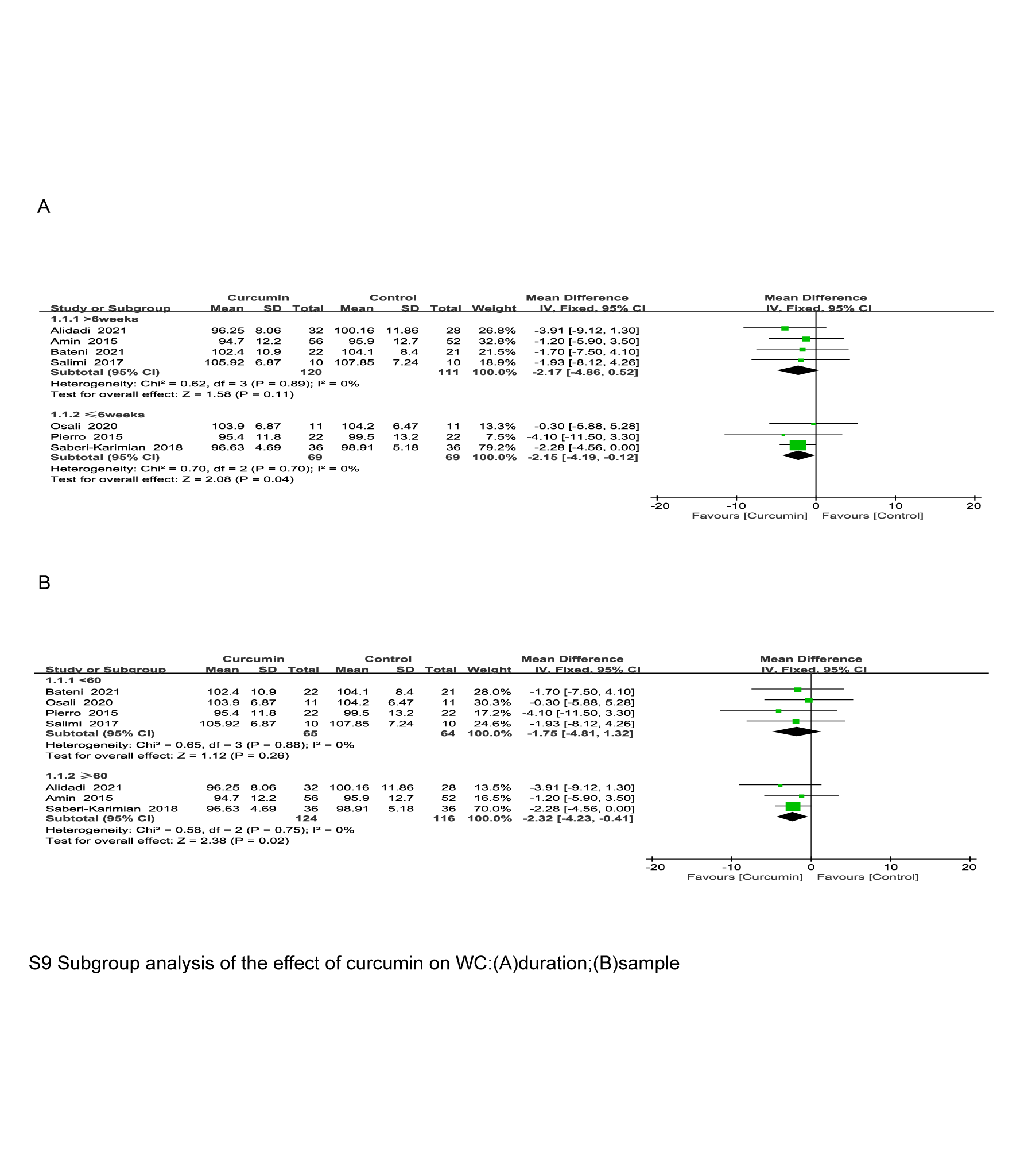

Supplement: Supplementary file 10 [file Image_9.tif]
